# Detecção de Hipercolesterolemia Familiar em Adolescentes: o Rastreamento Universal é Fundamental

**DOI:** 10.36660/abc.20250178

**Published:** 2025-05-06

**Authors:** Fernando Yue Cesena

**Affiliations:** 1 Instituto Dante Pazzanese de Cardiologia São Paulo SP Brasil Instituto Dante Pazzanese de Cardiologia, São Paulo, SP - Brasil

**Keywords:** Hipercolesterolemia, Adolescente, Programas de Rastreamento

Hipercolesterolemia familiar (HF) é um distúrbio genético caracterizado pela depuração prejudicada de partículas de lipoproteína de baixa densidade (LDL) do plasma para os hepatócitos, resultando em níveis elevados de colesterol LDL (LDL-C) no plasma e um risco significativamente aumentado de doença cardiovascular aterosclerótica (DCVA) prematura. Metanálises recentes indicam uma prevalência de HF de aproximadamente 1 em 310 indivíduos na população geral.^1 , 2^ É importante ressaltar que a HF continua substancialmente subdiagnosticada, e a maioria dos indivíduos afetados vive com níveis de LDL-C acima das metas recomendadas.^3^

A identificação precoce da HF é crítica para prevenir eventos de DCVA. De uma perspectiva fisiopatológica, a aterosclerose se desenvolve devido à exposição arterial cumulativa a partículas LDL elevadas. Tanto a magnitude de partículas LDL excessivas no plasma quanto a duração da exposição são relevantes para o desenvolvimento e progressão da placa.^4^ Nesse sentido, a HF é particularmente preocupante porque os indivíduos afetados têm níveis muito altos de LDL-C desde o nascimento/infância. O diagnóstico precoce e consequente conscientização do problema aumentam a chance de adoção de um estilo de vida saudável a longo prazo. A redução precoce do LDL-C melhora a função endotelial, atenua a progressão da aterosclerose e diminui o risco de eventos coronários.^3 , 5 , 6^

Nesta edição dos Arquivos Brasileiros de Cardiologia, um subestudo do ERICA, um estudo transversal brasileiro conduzido em 2013–2014, avalia anormalidades lipídicas sugestivas de HF em adolescentes de 12 a 17 anos da região metropolitana de Curitiba. Entre 2.383 adolescentes avaliados, 11 (aproximadamente 0,5% ou 1 em 217) apresentaram LDL-C >160 mg/dL ou colesterol não-HDL >190 mg/dL. O rastreamento em cascata foi aplicado a parentes de primeiro grau. Nenhum dos 7 alunos avaliados tinha diagnóstico de HF possível ou provável de acordo com a versão modificada da *Dutch Lipid Clinic Network* (Dutch MEDPED), enquanto 3 entre 15 parentes de primeiro grau (20%) tinham critérios para HF possível ou provável.^7^ O estudo tem limitações, incluindo a falta de um diagnóstico definitivo de HF por teste genético. Ainda assim, oferece uma oportunidade para discutir aspectos relevantes do diagnóstico de HF na juventude.

A ausência de adolescentes com critérios positivos para HF usando o Dutch MEDPED concorda com a recomendação contra o uso desta pontuação para diagnosticar HF em crianças e adolescentes.^5^ Esses indivíduos apresentam com menos frequência características típicas da HF, como xantoma tendinoso, em comparação aos adultos.^8^ Além disso, a DCVA clínica, um critério no Dutch MEDPED, ocorre tipicamente na vida adulta em indivíduos com HF heterozigótica. Portanto, a HF deve ser suspeita em crianças/adolescentes quando o LDL-C estiver acentuadamente elevado (>190 mg/dL não tratado) ou naqueles com LDL-C moderadamente elevado juntamente com histórico parental de doença arterial coronária prematura, LDL-C muito elevado ou uma mutação genética identificada causando HF.^3 , 5^

Testes genéticos para variantes causadoras de HF devem ser feitos sempre que possível.^3^ A identificação de uma variante patogênica indica um risco cardiovascular maior, melhora a adesão ao tratamento e pode levar ao rastreamento em cascata para identificar parentes afetados.^9 , 10^ No Brasil, o Hipercol Brasil ( https://hipercolbrasil.com.br/o-programa/ ), um programa de rastreamento genético em cascata da HF, exemplifica uma iniciativa bem-sucedida para facilitar o diagnóstico genético da HF mesmo em regiões distantes de centros especializados.^10 , 11^

No nível comunitário, o diagnóstico precoce da HF depende de uma estratégia de rastreamento eficaz, o que constitui um forte argumento a favor do rastreamento universal. Neste sentido, a Atualização da Diretriz Brasileira de HF de 2021 recomenda a realização do perfil lipídico para todas as crianças a partir dos 10 anos ou já a partir dos 2 anos em situações específicas (por exemplo, histórico parental de doença arterial coronária prematura ou LDL-C muito alto).^12^ A chance de detectar hipercolesterolemia em adolescentes não é irrisória. Uma publicação anterior do estudo ERICA mostrou que 25% das adolescentes do sexo feminino e 15% dos do sexo masculino tinham altos níveis de colesterol total plasmático (≥170 mg/dL), enquanto outros 26% das mulheres e 23% dos homens tinham colesterol total entre 150 mg/dL e 169 mg/dL, resultando em uma estimativa de quase 3 milhões de adolescentes brasileiros com colesterol sanguíneo elevado.^13^

A falta de uma estratégia estruturada para rastreamento lipídico universal na prática rotineira representa uma oportunidade perdida para redução precoce do risco cardiovascular por meio de intervenções no estilo de vida e terapias preventivas. Outras barreiras significativas permanecem, incluindo baixa conscientização sobre HF, especialmente em crianças/adolescentes, percepções errôneas do paciente em relação ao risco cardiovascular e baixa obtenção da meta de LDL-C em indivíduos com hipercolesterolemia grave.^14 , 15^ Abordar essas questões requer uma estratégia multifacetada envolvendo agências governamentais de saúde, sociedades médicas e grupos organizados de pacientes, com programas abrangentes e campanhas educacionais direcionadas a profissionais de saúde e à comunidade em geral. Essas ações são essenciais para reduzir efetivamente o impacto da HF e o risco de DCVA associado.
