# Supplementary material for: Detecção de Hipercolesterolemia Familiar em Adolescentes: o Rastreamento Universal é Fundamental
Source: Arq Bras Cardiol. 2025 May 6;122(3):e20250178. [Article in Portuguese] doi: 10.36660/abc.20250178 (PMC12101576; doi:10.36660/abc.20250178)
Supplement: Translation

# Translation: Detecting Familial Hypercholesterolemia in Adolescents: Universal Screening is Key

**DOI:** 10.36660/abc.20250178i

**Authors:** Fernando Yue Cesena

**Affiliations:** 1 São Paulo SP Brazil Instituto Dante Pazzanese de Cardiologia, São Paulo, SP - Brazil

**Keywords:** Hypercholesterolemia, Adolescent, Mass Screening

Familial hypercholesterolemia (FH) is a genetic disorder characterized by impaired clearance of low-density lipoprotein (LDL) particles from plasma to hepatocytes, resulting in elevated plasma LDL-Cholesterol (LDL-C) levels and a significantly increased risk of premature atherosclerotic cardiovascular disease (ASCVD). Recent meta-analyses indicate an FH prevalence of approximately 1 in 310 individuals in the general population.^1 , 2^Importantly, FH remains substantially underdiagnosed, and most affected individuals live with LDL-C levels above recommended targets.^3^

Early identification of FH is critical for preventing ASCVD events. From a pathophysiological perspective, atherosclerosis develops due to cumulative arterial exposure to elevated LDL particles. Both the magnitude of excessive LDL particles in plasma and the duration of exposure are relevant for plaque development and progression.^4^In this regard, FH is of particular concern because affected individuals have very high LDL-C levels since birth/childhood. Early diagnosis and consequent awareness of the problem increase the chance of adopting a healthy lifestyle in the long term. Early LDL-C lowering improves endothelial function, attenuates atherosclerosis progression, and diminishes the risk of coronary events.^3 , 5 , 6^

In this issue of *Arquivos Brasileiros de Cardiologia* , a substudy from ERICA, a cross-sectional Brazilian study conducted in 2013–2014, evaluates lipid abnormalities suggestive of FH in adolescents aged 12–17 from the Curitiba metropolitan region. Among 2,383 adolescents assessed, 11 (approximately 0.5% or 1 in 217) exhibited LDL-C >160 mg/dL or non-HDL cholesterol >190 mg/dL. Cascade screening was applied to first-degree relatives. None of the 7 students evaluated had a diagnosis of possible or probable FH according to the modified version of the Dutch Lipid Clinic Network (Dutch MEDPED), while 3 among 15 first-degree relatives (20%) had criteria for possible or probable FH.^7^The study has limitations, including the lack of a definitive diagnosis of FH by genetic test. Still, it offers an opportunity to discuss relevant aspects of FH diagnosis in the youth.

The absence of adolescents with positive criteria for FH using the Dutch MEDPED agrees with the recommendation against the use of this score for diagnosing FH in children and adolescents.^5^These individuals less frequently have typical FH features such as tendon xanthoma compared to adults.^8^Furthermore, clinical ASCVD, a criterion in the Dutch MEDPED, typically occurs in adult life in individuals with heterozygous FH. Therefore, FH should be suspected in children/adolescents when the LDL-C is markedly elevated (>190 mg/dL untreated) or in those with moderately elevated LDL-C alongside parental history of premature coronary heart disease, severely elevated LDL-C, or an identified genetic mutation causing FH.^3 , 5^

Genetic testing for FH-causing variants should be made whenever feasible.^3^Identifying a pathogenic variant indicates a higher cardiovascular risk, improves treatment adherence, and may prompt cascade screening to identify affected relatives.^9 , 10^In Brazil, the Hipercol Brasil ( https://hipercolbrasil.com.br/o-programa/ ), an FH cascade genetic screening program, exemplifies a successful initiative to facilitate FH genetic diagnosis even in regions distant from specialized centers.^10 , 11^

At the community level, early diagnosis of FH depends on an effective screening strategy, which constitutes a strong argument in favor of universal screening. Accordingly, the 2021 Update of the Brazilian Guideline for FH recommends lipid profiling for all children starting at age 10 or as early as age 2 in specific situations (e.g., parental history of premature coronary heart disease or very high LDL-C).^12^The chance of detecting hypercholesterolemia in adolescents is not negligible. A previous report from the ERICA study showed that 25% of female and 15% of male adolescents had high levels of plasma total cholesterol (≥170 mg/dL), while an additional 26% of females and 23% of males had total cholesterol between 150 mg/dL and 169 mg/dL, resulting in an estimate of almost 3 million Brazilian adolescents with elevated blood cholesterol.^13^

The lack of a structured strategy for universal lipid screening in routine practice represents a missed opportunity for early cardiovascular risk reduction through lifestyle interventions and preventive therapies. Other significant barriers remain, including low awareness of FH, especially in children/adolescents, patient misperceptions regarding cardiovascular risk, and low LDL-C target achievement in individuals with severe hypercholesterolemia.^14 , 15^Addressing these issues requires a multifaceted approach involving governmental health agencies, medical societies, and organized groups of patients, with comprehensive programs and educational campaigns targeting healthcare professionals and the broader community. These actions are essential to reduce FH burden effectively and associated ASCVD risks.
